# Drug level testing as a strategy to determine eligibility for drug resistance testing after failure of ART: a retrospective analysis of South African adult patients on second‐line ART

**DOI:** 10.1002/jia2.25501

**Published:** 2020-06-09

**Authors:** Lucas E Hermans, Kim Steegen, Rob ter Heine, Rob Schuurman, Hugo A Tempelman, Robert Moraba, Erik van Maarseveen, Monique Nijhuis, Taryn Pillay, Derryn Legg‐E’Silva, Tracy Snyman, Jonathan M Schapiro, David M Burger, Sergio Carmona, Annemarie MJ Wensing

**Affiliations:** ^1^ Virology Department of Medical Microbiology University Medical Center Utrecht (UMCU) Utrecht The Netherlands; ^2^ Wits Reproductive Health and HIV Institute (Wits RHI) University of the Witwatersrand Johannesburg South Africa; ^3^ Ndlovu Research Consortium Elandsdoorn South Africa; ^4^ Department of Molecular Medicine and Haematology Faculty of Health Sciences University of the Witwatersrand Johannesburg South Africa; ^5^ Department of Molecular Medicine and Haematology National Health Laboratory Service (NHLS) Johannesburg South Africa; ^6^ Department of Pharmacy Radboud Institute for Health Sciences Radboud University Medical Center Nijmegen The Netherlands; ^7^ Clinical Pharmacology UMCU The Netherlands; ^8^ Department of Chemistry University of the Witwatersrand Johannesburg South Africa; ^9^ National Health Laboratory Service (NHLS) Johannesburg South Africa; ^10^ National Hemophilia Center Sheba Medical Center Ramat Gan Israel

**Keywords:** antiretroviral treatment, treatment failure, HIV drug resistance, adherence, drug level testing, low‐ and middle‐income countries

## Abstract

**Introduction:**

When protease inhibitor (PI)‐based second‐line ART fails, guidelines recommend drug resistance testing and individualized third‐line treatment. However, PI‐resistant viral strains are rare and drug resistance testing is costly. We investigated whether less costly PI‐exposure testing can be used to select those patients who would benefit most from drug resistance testing.

**Methods:**

We performed a retrospective analysis of South African adults living with HIV experiencing failure of ritonavir‐boosted‐lopinavir (LPV/r)‐based second‐line ART for whom drug resistance testing results were available. We included patients who received plasma‐based drug resistance testing at a central South African reference laboratory in 2017 and patients who received dried blood spots (DBS)‐based drug resistance testing at a rural South African clinic between 2009 and 2017. PI‐exposure testing was performed on remnant plasma or DBS using liquid chromatography mass spectrometry (LCMS). Additionally, a low‐cost immunoassay was used on plasma. Population genotypic drug resistance testing of the *pol* region was performed on plasma and DBS using standard clinical protocols.

**Results:**

Samples from 544 patients (494 plasma samples and 50 DBS) were included. Median age was 41.0 years (IQR: 33.3 to 48.5) and 58.6% were women. Median HIV‐RNA load was 4.9 log10 copies/mL (4.3 to 5.4). Prevalence of resistance to the NRTI‐backbone was 70.6% (349/494) in plasma samples and 56.0% (28/50) in DBS. Major PI‐resistance mutations conferring high‐level resistance to LPV/r were observed in 26.7% (132/494) of plasma samples and 12% (6/50) of DBS. PI‐exposure testing revealed undetectable LPV levels in 47.0% (232/494) of plasma samples and in 60.0% (30/50) of DBS. In pooled analysis of plasma and DBS samples, detectable LPV levels had a sensitivity of 90% (84% to 94%) and a negative predictive failure of 95% (91% to 97%) for the presence of major LPV/r resistance.

**Conclusions:**

PI‐exposure testing revealed non‐adherence in half of patients experiencing failure on second‐line ART and accurately predicted the presence or absence of clinically relevant PI resistance. PI‐exposure testing constitutes a novel screening strategy in patients with virological failure of ART that can differentiate between different underlying causes of therapy failure and may allow for more effective use of limited resources available for drug resistance testing.

## 1 Introduction

Antiretroviral treatment (ART) programmes are expanding rapidly to people living with HIV in low‐ and middle‐income countries (LMIC) in an effort to control the HIV epidemic. First‐line ART treatment results in high on‐treatment virological suppression rates in these settings. [1, 2] In case of first‐line treatment failure, World Health Organization (WHO) guidelines recommend an empiric switch to a standardized second‐line regimen, consisting of a ritonavir‐boosted protease inhibitor (PI), either lopinavir (LPV/r) or atazanavir (ATV/r), combined with a dual nucleos(t)ide reverse transcriptase inhibitor (NRTI) backbone, with zidovudine and lamivudine as the recommended option after failure of TDF‐containing first‐line ART. [3] Second‐line ART in LMIC is currently prescribed to over 900.000 patients in LMIC and is predominantly LPV/r‐based. [4]

Despite the dosing complexity and potential toxicity of these regimens, several studies have reported good virological response to programmatic second‐line treatment in LMIC. [5, 6, 7] Notwithstanding this success, one‐third of patients does not achieve virological suppression. [8] In case of failure WHO guidelines recommend drug resistance testing to establish the presence of PI drug resistance and selection of a third‐line regimen based on the drug resistance profile. However, studies have shown that PI‐resistant viral strains are only detected in 17% to 36% of these patients, suggesting that in most cases failure is merely due to non‐adherence without evident selection of drug resistance. [9, 10, 11]

Drug resistance testing is costly, has a long turn‐around time, and requires extensive laboratory infrastructure and specialist interpretation. Due to these factors, capacity for resistance testing is limited in most LMIC. [12] The low prevalence of PI resistance implies that where resistance testing is implemented, PI resistance is only encountered in one in four patients with virological failure on second‐line ART. Given the limited resources, targeted use of drug resistance testing in patients with established adherence to medication would potentially increase efficiency and reduce costs.

Accurate screening tests for adherence to treatment would enable preselection of adherent patients for drug resistance testing. Moreover, this approach would also provide a point of entry into intensified adherence counselling for non‐adherent patients. While currently used surrogate adherence measures such as patient‐reported adherence and pharmacy refill data generally correlate with treatment outcomes, these tests are either subjective or vulnerable to manipulation. [13, 14, 15, 16, 17] Hence, objective markers to determine adherence to second‐line ART are urgently required.

We studied the value of recent PI‐exposure measured by qualitative plasma LPV level testing as an objective adherence marker and screening tool for PI drug resistance in patients with virological failure on second‐line ART. We report the accuracy of this novel treatment monitoring strategy on plasma samples as well as on dried blood spots (DBS), the predominant sample types in use for HIV‐RNA viral load and HIV drug resistance testing.

## 2 methods

### 2.1 Study design

This is a retrospective analysis of adult (≥18 years) people living with HIV attending public healthcare facilities. Patients were included if (1) they were receiving treatment with LPV/r‐based second‐line ART, (2) if they experienced confirmed virological failure according to South African guidelines, defined as viraemia ≥1000 copies/mL for at least six months, after at least one year of PI‐based second‐line ART, [18] and (3) if drug resistance was requested and successfully performed in clinical practice.

This study was conducted with ethical clearance from the Research on Human Subjects (Medical) Committee at the University of the Witwatersrand (Clearance certificate number M180332). In accordance with the Committee policy, no informed consent was obtained from the patients due to the retrospective nature of the study. No additional samples were taken from patients for the purposes of this study.

### 2.2 Study procedures

Plasma samples were analysed at the National Health Laboratory Services (NHLS) laboratory at Charlotte Maxeke Johannesburg Academic Hospital, Johannesburg, South Africa, a referral laboratory for HIV drug resistance testing that services public healthcare facilities across four provinces, covering approximately 40% of the South African national treatment programme. Out of a total of 786 eligible patients who received HIV drug resistance testing in 2017 for virological failure on second‐line ART, a computer‐generated random selection of 500 patients was made. Patients received drug resistance testing on EDTA‐derived plasma as part of clinical care. Remaining plasma was stored at a temperature monitored facility at −80°C. Drug exposure testing was performed batchwise on these stored samples.

In addition, to demonstrate the feasibility of the approach using alternative sampling materials, the study was also implemented at a large rural primary healthcare facility based in Limpopo, South Africa, which provides ART to a large and well‐described cohort of over 3600 people living with HIV. Between December 2009 and August 2017, 50 patients with virological failure on second‐line ART received DBS‐based genotypic drug resistance testing as part of routine clinical management. Per patient, five drops of 50 µL of EDTA‐derived whole blood were spotted on a Protein Saver 903 card (Whatman Nederland B.V., Den Bosch, The Netherlands) and were left to dry overnight prior to packaging in a zip‐lock bag containing desiccant. Samples were shipped on room temperature to the Translational Virology Laboratory of the University Medical Center Utrecht (UMCU), Utrecht, The Netherlands, where all analyses were performed. Drug resistance testing was performed within three weeks of sample collection using two spots as input and results were reported back to the treating clinician. The remaining spots were subsequently stored at a temperature monitored facility at −20°C. Drug exposure testing was performed batchwise on thawed DBS, using one spot as input.

### 2.3 Plasma and DBS genotypic drug resistance testing

Population‐based genotypic drug resistance testing was performed using previously described methods for plasma [19] and for DBS. [20]

Drug resistance to LPV/r was defined as the presence of at least one of the following IAS‐USA defined LPV/r resistance‐associated mutations in protease: V32I, I47V/A, I50V, I54V/L/M, L76V, V82A/F/T/S and I84V. [21] Drug susceptibility scores were calculated using the Stanford database. Sequences were submitted to Genbank (accession number # 2343427).

### 2.4 Plasma and DBS drug exposure testing

PI‐exposure testing was primarily performed using high performance liquid chromatography‐tandem mass spectrometry (LCMS).

For plasma‐based drug level testing, LCMS was performed on EDTA‐derived plasma using a validated clinical protocol for quantitative plasma drug level measurement that is subjected to regular external quality assurance. [22] For LPV, the assay was linear (R^2^ = 0.995) in the range of 0.03 to 11.16 mg/L. The coefficient of variance of low and high quality control samples (mean concentrations of 2.34 and 8.10 mg/L respectively) was below 10%. Plasma LPV levels above the assay limit of detection (LOD) of 0.01 mg/L were considered detectable. Screening for detectable levels of other antiretroviral anchor drugs (atazanavir, darunavir, efavirenz, nevirapine and raltegravir) was also performed to exclude any unreported use of other antiretroviral drugs.

In addition to LCMS‐based testing, PI‐exposure testing was repeated on a rapid automated enzyme immunoassay (EIA) for detection of lopinavir (ARK Diagnostics Inc, Fremont, California, USA) that was implemented on the Indiko™ Plus benchtop chemistry system (Thermo‐Scientific, Waltham, MSA, USA). Assay validation is described elsewhere. [23] Plasma LPV levels above the assay LOD of 0.04 mg/L were considered detectable.

For DBS‐based testing, an in‐house LCMS procedure optimized for the detection of LPV in DBS was used. The assay proved linear (R^2^ = 0.98) in the range of 0.25 mg/L to 8.00 mg/L, with quality control sample results (low, medium and high) within 20% of the reference range. Plasma LPV concentrations were estimated from dried blood spot LPV concentrations according to a previously validated formula: DBS concentration/(1‐haematocrit). [24] Estimated plasma LPV concentrations above the LOD of 0.25 mg/L were considered detectable.

### 2.5 Statistical analysis

Diagnostic accuracy of LPV exposure testing as a marker for LPV/r resistance was reported as sensitivity, specificity, and positive/negative predictive value (PPV/NPV). Accuracy at varying threshold levels was assessed using receiver‐operated characteristic (ROC) analysis. For ROC analysis, levels above the upper limit of quantification (LOQ) and below the lower LOQ were censored at the respective LOQ. Univariate comparisons were performed using the unpaired Student’s *t*‐test or Mann‐Whitney *U*‐test for continuous data, and χ*^2^*‐test or Fisher’s exact test for categorical data. Multivariable analysis of predictors of LPV/r‐resistance was performed using logistic regression. Age, sex, second‐line NRTI‐backbone, HIV‐RNA load at second‐line failure and study group (DBS vs. plasma) were entered as covariables. In a sensitivity analysis, stepwise backward selection of covariables to the models based on Akaike information criterion was used. The randomization list for the random sample of patient plasma samples was generated using stratified random sampling without replacement, where strata reflect the proportional sample contribution of each province to the dataset. All statistical data analysis was performed using *R* version 3.4.1 (The R foundation for statistical computing) and *Rstudio* version 1.0.153, the *epiR* (version 0.9‐99) and *pROC* (version 0.13.0) statistical packages, and the publicly available *stratified* function (https://www.rdocumentation.org/packages/fifer/versions/1.0/topics/stratified) for random sampling.

## 3 results

### 3.1 Patient characteristics

From 500 patients selected for plasma‐based testing, 494 patients were included, originating from 72 healthcare facilities (see supplementary materials). Fifty patients were selected for DBS‐based testing and all were included.

All 544 included patients had documented virological failure of LPV/r‐based second‐line ART. All patients received LPV/r with a dual NRTI backbone. 58.6% (319/544) of patients were female, with a median age of 41.0 years (interquartile range (IQR): 33.3 to 48.5). Median HIV‐RNA load at time of failure was 4.9 log copies/mL (IQR: 4.3 to 5.4). Patients were infected with HIV‐1 subtype C in 98.9% (538/544) of cases. (Table 1).

**Table 1:** Patient characteristics

Patient characteristics	Overall (n = 544 patients)	DBS group (n = 50 patients)	Plasma group (n = 494 patients)	*p*‐value
Sex (% female)	58.6% (319)	52.0% (26)	59.3% (293)	0.40
Age (years)	41.0 [33.3 to 48.5]	40.5 [33.5 to 50.4]	41.1 [33.2 to 48.3]	0.98
Log HIV‐RNA at second‐line failure (log copies/mL)	4.9 [4.3 to 5.4]	4.7 [3.8 to 5.1]	4.9 [4.4 to 5.4]	0.03
Second‐line NRTI backbone				0.68
AZT/3TC	62.1% (338)	70.0% (35)	61.3% (303)	
TDF/3TC	18.9% (103)	14.0% (7)	19.4% (96)	
ABC/3TC	13.8% (75)	12.0% (6)	14.0% (69)	
Other	5.1% (28)	4.0% (2)	5.3% (26)	
HIV‐1 subtype (% subtype C)	98.9% (538)	100% (50)	98.8% (488)	0.94
Time since ART start (months)	NA	65.3 [40.4 to 84.3]	NA	NA
Time since start second‐line ART (months)	NA	32.1 [17.9 to 56.0]	NA	NA
CD4‐count at start ART (cells/mm^3^)	NA	82 [46 to 191]	NA	NA
CD4‐count at start second‐line ART (cells/mm^3^)	NA	216 [78 to 352]	NA	NA

Results displayed as percentage (count) or median [interquartile range]. Time on treatment and CD4‐count were not assessed in the plasma group. *p*‐values for differences calculated using χ^2^‐test for categorical variables and Mann‐Whitney Wilcoxon for continuous variables. 3TC, lamivudine; ART, Antiretroviral treatment; ABC, abacavir; AZT, zidovudine; CD4‐count, CD4+ T‐lymphocyte count; DBS, Dried Blood Spot; NRTI, nucleos(t)ide reverse transcriptase inhibitor; TDF, tenofovir disoproxil fumarate.

### 3.2 Drug resistance and PI‐exposure testing

**Figure 1:**
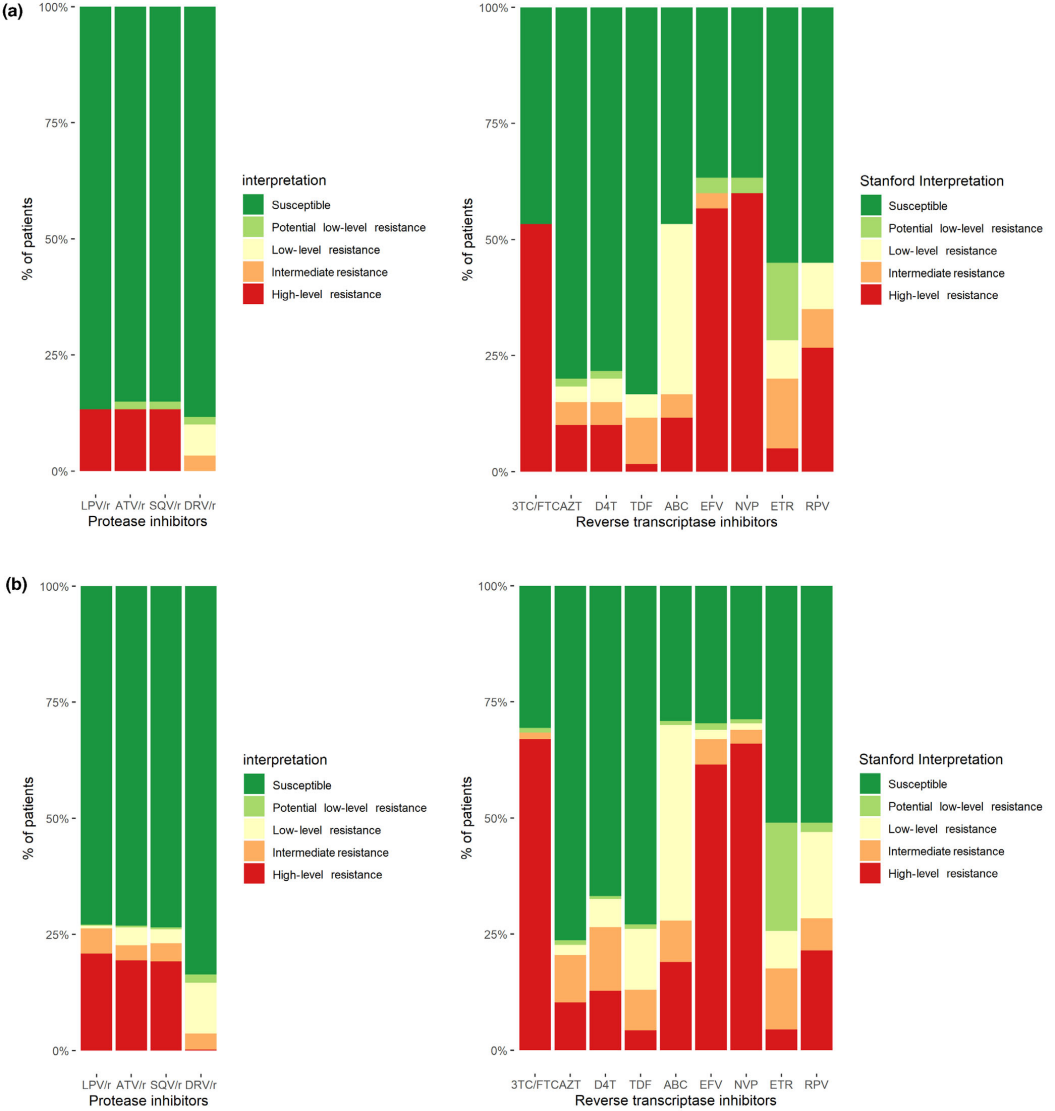
**A**, Drug susceptibility in DBS group (n = 50 patients). **B**, Drug susceptibility in plasma group (n = 494 patients). Proportion of patients with resistance to antiretroviral compounds according to the Stanford HIV drug resistance database. 3TC/FTC, lamivudine/emtricitabine; ABC, abacavir; ATV, atazanavir; AZT, zidovudine; D4T, stavudine; DBS, Dried‐Blood Spot; DRV, darunavir; EFV, efavirenz; ETR, etravirine; LPV, lopinavir; NVP, nevirapine; r, ritonavir; RPV, rilpivirine; SQV, saquinavir; TDF, tenofovir disoproxil fumarate.

Mutations conferring resistance to NNRTIs were detected in in 64.2% (317/494) of plasma samples and in 54.0% (27/50) of DBS. Mutations conferring resistance to NRTIs were detected in 70.6% (349/494) of plasma samples and 56.0% (28/50) of DBS. LPV/r‐resistant viral strains were only detected in 26.7% (132/494) of plasma samples and 12.0% (6/50) of DBS (Figure 1).

In plasma samples, PI‐exposure testing revealed undetectable LPV levels in 47.0% (232/494) of patients using LCMS and 45.4% (221/487) of patients using EIA. For LCMS, sensitivity of LPV level for LPV/r resistance was 89% (95% CI: 83% to 94%), and NPV was 94% (90% to 97%). For EIA, sensitivity of LPV level for LPV/r resistance was 88% (82% to 93%), and NPV was 93% (89% to 96%) (Table 2). In DBS, PI‐exposure testing revealed undetectable LPV levels in 60.0% (30/50) of patients. Prevalence of LPV/r resistance in patients with undetectable LPV levels was 0.0% (0/30), versus 30.0% (6/20) in patients with detectable LPV levels. The sensitivity of LPV level for LPV/r resistance was 100% (95% CI: 54% to 100%) and NPV was 100% (88% to 100%) (Table 2).

**Table 2:** Diagnostic accuracy of detectable LPV level for LPV/r‐resistance

	DBS group (n = 50 patients)	Plasma group (n = 494 patients)		Combined results (n = 544 patients)
	DBS‐LCMS	plasma‐LCMS	plasma‐EIA	DBS‐LCMS & plasma‐LCMS
Sensitivity	100% [54 to 100]	89% [83 to 94]	88% [82 to 93]	90% [84 to 94]
Specificity	68% [52 to 81]	60% [55 to 65]	58% [52 to 63]	61% [56 to 66]
Positive predictive value	30% [12 to 54]	45% [39 to 51]	43% [37 to 49]	44% [38 to 50]
Negative predictive value	100% [88 to 100]	94% [90 to 97]	93% [89 to 96]	95% [91 to 97]

Diagnostic accuracy of qualitative detection of LPV level for LPV/r‐resistance. Levels of detection were 0.25 mg/L for the DBS‐LCMS, 0.01 mg/L for the plasma‐LCMS, and 0.04 mg/L for the plasma‐EIA. Results displayed as percentage [95% confidence interval]. DBS, Dried Blood Spot; EIA, Enzyme Immunoassay; LCMS, Liquid chromatography‐tandem mass spectrometry.

In combined analysis of plasma and DBS results (n = 544), detectable LPV had a high sensitivity of 90% (84% to 94%) and NPV of 95% (91% to 97%) for LPV/r resistance (Table 2). A detectable LPV level also predicted presence of the M184V resistance mutation conferring resistance to 3TC/FTC which may rapidly disappear from detection in the plasma in case of non‐adherence. In patients with detectable LPV the prevalence of M184V was 50.4% (132/262) versus 20.6% (58/282) in patients with undetectable LPV (*p* < 0.001). Patients with detectable LPV also had a significantly lower plasma HIV‐RNA load when compared to patients with undetectable LPV (4.7 vs. 5.0 log copies/mL; *p* < 0.001).

**Figure 2:**
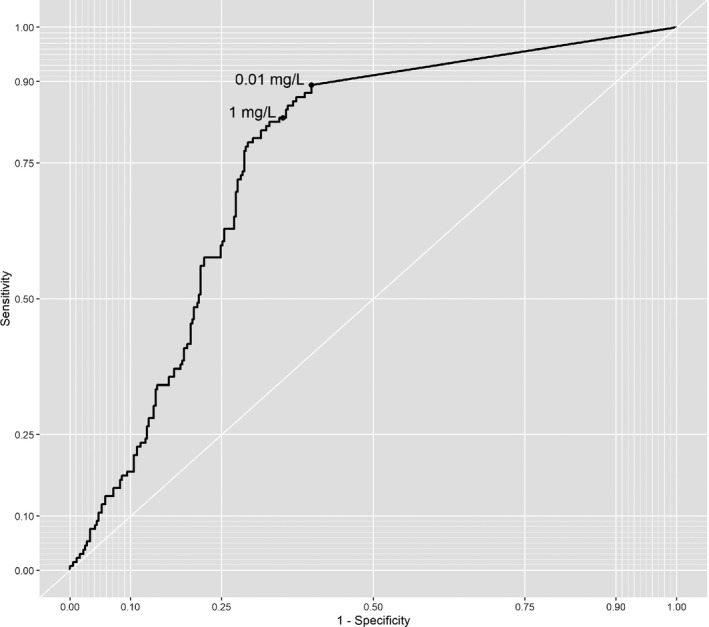
Receiver‐operated characteristic (ROC) curve. ROC curve of the diagnostic accuracy of lopinavir drug levels measured by liquid chromatography‐mass spectrometry (LCMS) for LPV/r resistance. Thresholds are set at the limit of detection of the LCMS assay (0.01 mg/L) and the minimum recommended lopinavir trough level (1 mg/L).

Assessment of different LPV detection thresholds in quantitative testing performed on plasma samples revealed that sensitivity was 83% (76% to 89%) at the minimum recommended LPV trough level of 1 mg/L. In ROC analysis, LPV drug level had an area under the curve of 75.6% for prediction of LPV/r resistance (95% CI: 71.2% to 80.0%). Optimal sensitivity was attained at a threshold of 0.01 mg/L, confirming the benefit of a low threshold for application as a screening test (Figure 2).

In fourteen cases an undetectable LPV level was encountered in the presence of LPV/r resistance. In‐depth analysis of viral genetic profiles revealed that when compared to cases of LPV/r resistance with detectable LPV levels, major PI drug resistance mutations were more likely present in mixtures with wild type (42.9% (6/14) vs. 16.9% (20/118); Fisher’s exact *p* = 0.032), and M184V was less prevalent (78.6% (11/14) versus 94.9% (112/118); Fisher’s exact *p* = 0.055).

### 3.3 Drug resistance profiles

Of predefined LPV/r resistance mutations, a single LPV/r resistance mutation was detected in 16.7% (23/138), two mutations in 42.8% (59/138) and three or more in 40.6% (56/138) of cases in combined analysis of plasma and DBS results (Table 3). The majority of patients harboured either I54V + V82A or I54V + L76V + V82A (Table 3). Cross‐resistance to darunavir/ritonavir as interpreted using Stanford susceptibility scores was present in 55.1% (76/138) of cases, with 41.3% (57/138) harbouring low‐level, 13.0% (18/138) intermediate and 0.7% (1/138) high‐level resistance. The most prevalent darunavir resistance‐associated mutations were L76V in 42.0% (58/138), L33F in 15.9% (22/138) and I84V in 8.7% (12/138) of cases. Two or more darunavir resistance‐associated mutations were present in 13.8% (19/138) of cases.

**Table 3:** Protease mutational patterns (n = 138)

Bold LPV/r mutations	Non‐bold LPV/r mutations	Frequency, %	n
V32I + I47A	–	1.4	2
V32I + I47A + I50V	L33F + M46I	0.7	1
I47A	M46L + F53L	0.7	1
I47A	L10F + L24I + M46I	0.7	1
I47A + I84V	–	0.7	1
I47A + I54V + V82A	L10F + M46L	0.7	1
I47A/T/V + I54V + V82A	L10F + L24I + M46I	0.7	1
I47V + I54V + L76V	M46I	0.7	1
I50V	–	0.7	1
I50V + I54V + V82A	L10F + L33F	0.7	1
I50V + I54V	M46I	0.7	1
I50V + I54V + V82A	L33F + M46I	0.7	1
I54V	–	1.4	2
I54V	L10F	0.7	1
I54V	M46L	0.7	1
I54V + L76V	–	0.7	1
I54V + L76V	M46I	1.4	2
I54V + L76V + V82A	–	0.7	1
I54V + L76V + V82A	L10F	0.7	1
I54V/L + L76V + V82A/F	M46I	7.2	10
I54V + L76V + V82A	L10F/V + M46I/L	18.1	25
I54V + L76V + V82A	L33F + M46I	1.4	2
I54V + L76V + V82A	M46I + F53L	0.7	1
I54V + L76V + V82A	L10F + L33F + M46I	2.9	4
I54V + L76V + V82A	L10F + L24I + L33F + M46I	1.4	2
I54V + L76V + I84V	M46I	0.7	1
I54V + L76V + I84V	M46I + L10F	0.7	1
I54V + V82A	–	4.3	6
I54V + V82A	L10F	0.7	1
I54V + V82A	M46I/L	6.5	9
I54V + V82A	L10F/V + M46I/L	11.6	16
I54V + V82A	L24I + M46I	1.4	2
I54V + V82A	M46L + L90M	0.7	1
I54V + V82A	L24I + L33F + M46L	0.7	1
I54V + V82A	L10F + L24I + M46I	0.7	1
I54V + V82A/S	L10F + L33F + M46I	4.3	6
I54V + V82A	L10F + L24I + L33F + M46I/L	2.9	4
I54V + V82A + I84V	L10F + M46I	0.7	1
I54V + I84V	–	0.7	1
I54V + I84V	L10F	0.7	1
I54V + I84V	L10F + L24I + M46I	0.7	1
L76V	L10F	0.7	1
L76V	M46I	1.4	2
L76V + I84V	L10F + M46I	1.4	2
L76V + V82A + I84V	M46I	0.7	1
V82A	–	5.1	7
V82A	L10F	1.4	2
V82A	M46I/L	1.4	2
I84V	M46I + L10F	1.4	2

Frequency of mutational patterns in protease detected by Sanger sequencing conferring ritonavir‐boosted lopinavir (LPV/r) drug resistance in 138 patients with virological failure on LPV/r‐based second‐line ART. Bold and non‐bold mutations specified according to IAS‐USA figures. [21] Amino acid substitutions either complete or in mixture with wild type were combined.

In multivariable logistic regression older age, likely reflecting duration of treatment, was associated with a higher likelihood of harbouring LPV/r‐resistant virus (42.7 vs. 40.4 years, aOR 1.21 (1.10 to 1.32), *p* < 0.001). A trend of less LPV/r resistance in patients on non‐standard backbones versus AZT/3TC‐backbone was observed, but this was not statistically significant (*p* = 0.067). In the DBS group we observed that patients with LPV/r‐resistant virus had a longer median duration of second‐line ART (64.4 (36.2 to 75.7) vs. 30.8 (16.2 to 51.7) months), a trend that bordered on statistical significance (Mann‐Whitney *p* = 0.052). HIV‐RNA load and sex were not significantly correlated with LPV/r resistance (Table 4). Stepwise backward model selection did not substantially change the multivariable analysis results (supplementary materials).

**Table 4:** Characteristics of LPV/r resistance versus no LPV/r resistance

Patient Characteristics	No LPV/r resistance (n = 406)	LPV/r resistance (n = 138)	Univariate		Multivariate	
			OR [IQR]	*p*‐value	aOR [IQR]	*p*‐value
Sex (% female)	59.1% (240)	57.2% (79)	0.93 [0.63 to 1.37]	0.78	1.03 [0.68 to 1.56]	0.89
Age (years)	40.4 [31.2 to 48.1]	42.7 [37.0 to 50.7]	1.19 [1.09 to 1.30]	<0.001	1.21 [1.10 to 1.32]	<0.001
Log HIV‐RNA at second‐line failure (log copies/mL)	4.9 [4.3 to 5.4]	4.9 [4.5 to 5.4]	1.18 [0.93 to 1.51]	0.28	1.23 [0.95 to 1.59]	0.11
Second‐line NRTI backbone						
AZT/3TC	60.3% (245)	67.4% (93)	Ref		Ref	
TDF/3TC	18.7% (76)	19.6% (27)	0.94 [0.56 to 1.53]	0.80	0.97 [0.57 to 1.61]	0.91
ABC/3TC	14.8% (60)	10.9% (15)	0.66 [0.35 to 1.19]	0.18	0.65 [0.33 to 1.19]	0.17
Other	6.2% (25)	2.2% (3)	0.32 [0.07 to 0.93]	0.06	0.32 [0.07 to 0.94]	0.07
Time since ART start (months)	64.9 [39.9 to 81.0]	105.9 [67.3‐ to 137.3]	1.02 [1.00 to 1.05]	0.14	NA	NA
Time since start second‐line ART (months)	30.8 [16.2 to 51.7]	64.4 [36.2 to 75.7]	1.04 [1.00 to 1.08]	0.05	NA	NA
CD4‐count at start ART (cells/mm^3^)	85 [47 to 190]	38 [33 to 115]	0.83 [0.31 to 1.18]	0.54	NA	NA
CD4‐count at start second‐line ART (cells/mm^3^)	192 [75.333]	327 [288 to 393]	1.14 [0.91 to 1.41]	0.05	NA	NA

Results displayed as percentage (count) or median [interquartile range]. Time on treatment and CD4‐count were not assessed in the plasma group. OR for differences calculated using χ^2^‐test for categorical variables and Mann‐Whitney Wilcoxon for continuous variables. aOR for LPV/r resistance was calculating using multivariable logistic regression, entering sex (reference = male), age (per five years increment), log HIV‐RNA (per 1 log increment), and NRTI‐backbone (reference = AZT/3TC) as covariables and correcting for sampling group (DBS vs plasma). Time on treatment was assessed in months and CD4‐count in increments of 50 cells/mm^3^. These variables were only available for patients in the DBS group and therefore not entered in multivariable analysis. 3TC, lamivudine; ART, Antiretroviral treatment; ABC, abacavir; aOR, adjusted Odds Ratio; AZT, zidovudine; CD4‐count, CD4+ T‐lymphocyte count; DBS, Dried Blood Spot; IQR, interquartile range; LPV/r, ritonavir‐boosted lopinavir; NRTI, nucleos(t)ide reverse transcriptase inhibitor; TDF, tenofovir disoproxil fumarate.

## 4 Discussion

In this largest‐to‐date analysis of patients with virological failure on second‐line ART in LMIC, LPV plasma levels were not detected in half of patients. An undetectable LPV level excluded the presence of PI‐resistant viral strains with a high degree of certainty. Results were consistent between DBS‐based testing in a rural healthcare facility and plasma‐based testing at a reference laboratory.

In this analysis, only a minority of patients with virological failure harboured PI‐resistant virus. This finding is in line with previous observations from LMIC. [8] The low prevalence of PI‐resistance may be explained by the high genetic barrier to resistance of boosted PI‐based ART, but is also suggestive of significant non‐adherence, as highlighted by the high proportion of patients with undetectable LPV levels in this study. Non‐adherence may be in part due to LPV/r‐related side effects. In particular, gastrointestinal side‐effects such as diarrhoea, nausea and vomiting are often reported by patients and are a leading cause for discontinuation of the drug. [25, 26] In addition, it has been suggested that empirical switching from first‐line to second‐line ART may fail to address socio‐economic or behavioural issues that affect adherence, which then persist during second‐line ART. [27]

The low prevalence of PI resistance necessitates further investigation of the underlying cause of failure, which is currently based solely on drug resistance testing. Population‐based drug resistance testing can be unreliable in case of non‐adherence, as drug sensitive strains may reemerge as the dominant viral population in plasma under these circumstances. [28, 29, 30] Assessment of adherence in current clinical practice is commonly based on patient‐reported adherence and pharmacy refill data. Patient‐reported adherence is inherently subjective and tends to overestimate adherence. [13, 14, 15, 16] While pharmacy refill data are a more objective measure of adherence, it requires patients to bring their medication to clinic visits and cannot account for patients receiving medication from multiple sources. [17] Previous attempts to achieve objective adherence assessment mainly focused on therapeutic drug monitoring (TDM), consisting of quantitative measurement of drug trough concentrations. This approach has been shown not to be of additional benefit over routine viral load monitoring. [31] Hence, routine TDM is currently not recommended in clinical guidelines. [3, 32]

Qualitative testing for recent drug exposure in patients with virological failure of second‐line ART constitutes a novel approach to treatment monitoring in resource‐limited settings. Implementation of this approach would allow for targeted use of expensive resistance testing in patients at high risk of harbouring PI‐resistant viral strains. If PI‐exposure testing would have been used to determine eligibility for drug resistance testing in this study, approximately half of all requested drug resistance tests could have been avoided. The routine implementation of such a two‐tiered approach holds the potential to simplify clinical management of patients experiencing virological failure.

Our study further demonstrates that this approach can be implemented in various different settings. The use of DBS facilitates implementation of drug exposure testing in settings where laboratory infrastructure and cold‐chain transport are unavailable and allows for subsequent drug resistance testing on the same DBS card. Moreover, successful use of a low‐cost immunoassay on plasma samples in this study shows that PI‐exposure testing can also be decentralized to settings with limited laboratory infrastructure. Immunoassays are easily implemented on standard chemistry analyzers at a fraction of the cost of drug resistance testing, and enable rapid feedback of results. The development of point‐of‐care (POC) technology for viral load measurement and drug resistance testing is set to change current ART monitoring algorithms. The recent development of POC tests for the detection of antiretroviral drugs in urine may pave the way for future implementation of drug level testing in POC ART monitoring strategies. [33, 34] More research is needed to assess the efficacy and cost‐efficiency of potential new ART monitoring strategies, including novel methodologies such as ART exposure testing. ART exposure testing could be of particular relevance to patients on dolutegravir‐containing first‐line ART. DTG has a high barrier to resistance, and viraemia on DTG‐containing ART is therefore very likely to be due to underlying non‐adherence in the absence of drug resistance to DTG.

To our knowledge, this is the first demonstration of diagnostic accuracy of qualitative PI‐exposure testing in patients with viraemia during PI‐based second‐line ART. We identified one previous study which retrospectively assessed quantitative lopinavir plasma concentrations and LPV/r‐resistance in 134 adult and paediatric patients experiencing failure of PI‐based second‐line ART. [35] Accuracy at the minimum lopinavir trough level of 1 mg/L was assessed, yielding a relatively low sensitivity for LPV/r drug resistance of 79%. In our study, the assay was not used to assess whether LPV plasma levels were therapeutic, but to qualitatively detect any recent PI exposure above the limit of detection of the assay. [36] We demonstrate that this approach has a much higher sensitivity and negative predictive value for LPV/r resistance. While false‐positive misclassification regularly occurred, false‐negative misclassification was rare, resulting in better screening test performance.

A small number of patients harboured LPV/r‐resistant virus in the absence of detectable LPV levels. The misclassification of these patients by PI exposure testing is likely indicative of intermittent non‐adherence or recent interruption of treatment. After treatment interruption, LPV levels will drop below detectable levels within one or two days, [37] while PI‐resistant viral strains may still be detectable with population‐based sequencing for several weeks to months. [28] The resistance patterns encountered in these patients were also suggestive of suboptimal adherence. PI resistance mutations were more frequently present in mixture and the M184V mutation, which is known to disappear rapidly after cessation of 3TC/FTC, [28] was more frequently absent. This implies that the full extent of drug resistance in these patients may not have been uncovered by drug resistance testing due to suboptimal adherence. While these patients do ultimately benefit from a switch to third‐line ART, the selection of this regimen needs to be informed by a reliable drug resistance testing result. We therefore argue that even though these cases of PI resistance were incorrectly classified by PI‐exposure testing, PI‐exposure testing would still constitute a useful screening test for these patients and would potentially enable optimisation of adherence prior to drug resistance testing.

More sensitive next‐generation sequencing techniques may be able to detect PI‐resistant viral subpopulations below the detection threshold of population‐based sequencing. The impact of these minority variants on treatment success has not been conclusively established, but is an area of ongoing research interest. [38] Next‐generation sequencing was not performed in this study, as the presence of drug‐resistant minority variants is currently not used to guide clinical decision making.

In patients with PI resistance, mutational patterns of PI resistance were extensive. This likely reflects the long duration of virological failure on PI‐based ART, which is inherent to annual viral load testing, and may be further aggravated by long turn‐around times and limited access to drug resistance testing and third‐line treatment. Accumulation of PI drug resistance mutations resulted in cross‐resistance to darunavir, a crucial component of third‐line regimens, in a substantial amount of cases. These findings further highlight the need for novel strategies to enable fast and informed decision‐making in case of failure on second‐line ART.

This study has several important limitations. Data on treatment history and duration were not available for patients in the plasma sample group. These factors may have explained the difference in prevalence of PI resistance between the plasma and DBS group. While the study results can likely be generalized to other Southern African countries, they may be less relevant to settings where different second‐line regimens, notably atazanavir‐based regimens are used. In addition, the results may be less applicable to settings were different HIV subtypes predominate or where patients were previously exposed to unboosted PI treatment. Strengths of this study include the use of samples obtained in routine clinical practice at various levels of healthcare, enabling a realistic assessment of the accuracy of PI exposure testing in standard clinical care. The use of routine clinical data also enabled us to perform the largest analysis to date of PI drug resistance prevalence in patients expressing virological failure of programmatic LPV/r‐based second‐line ART and to identify the most prevalent resistance pathways in HIV‐1 subtype C.

## 5 Conclusion

Although access to ART is rapidly scaling up in LMIC, access to laboratory monitoring is lagging behind. Novel diagnostic approaches using simple and low‐cost technologies may bridge this gap. This study demonstrates that PI‐exposure testing has the potential to improve and simplify clinical management in case of virological failure, by directing rationalized drug resistance testing in patients failing second‐line ART. Implementation of this strategy should be considered to enable more efficient use of limited resources.

## Competing Interest

The authors report no conflicts of interest.

## Authors’ Contributions

AMJW, SC, HAT, RS and LEH designed the study. LEH, RH, EM, MN, TP, DL, TS, AMJW and DMB were involved in drug level testing experiments. KS, RS, SC, and AMJW were involved in drug resistance testing experiments. RM, LEH and HAT were involved in the clinical care of patients in the study. LEH performed the statistical analyses. JMS, DMB and AMJW gave feedback on the analyses and reviewed preliminary results. LEH and AMJW drafted the first version of the manuscript. All authors provided feedback on the manuscript. All authors have read and approved the final manuscript.

## Supporting information


**Data S1**. Random selection of participants in the plasma group.
**Table S1**. Breakdown of patients in study per facility type and province.
**Table S2**. Stepwise backward automatic variable selection.
**Figure S1**. **A‐C**, Linearity of continuous variables against the logit.
