# Supplementary material for: Drug level testing as a strategy to determine eligibility for drug resistance testing after failure of ART: a retrospective analysis of South African adult patients on second‐line ART
Source: J Int AIDS Soc. 2020 Jun 9;23(6):e25501. doi: 10.1002/jia2.25501 (PMC7282495; doi:10.1002/jia2.25501)

# Supplementary Materials

Random selection of participants in the plasma group

**Exclusion:** Records were available for **932** unique patients who received drug resistance testing in 2017 at the NHLS laboratory at Charlotte Maxeke Johannesburg Academic Hospital, and who originated from Gauteng Province, North-West Province, Mpumalanga Province, or Limpopo Province. Of these, **106** were excluded based their clinical record. Exclusions were due to unavailable HIV-RNA load result (2), a last HIV-RNA load <1000 copies/mL (30), having received drug resistance testing for third-line ART failure (2), and being on a regimen containing either ATV/r or EFV as the base drug, and not LPV/r (72). For the resulting **826** patients, remnant sample material was sought. Remnant sample was retrieved for **786** patients.

**Random selection:** Of the **786** patients a random selection of 500 patients was made. For these patients, samples were retrieved and subjected to drug level testing. Six further patients were excluded during this process. Two patients were excluded as the remnant sample was insufficient for testing. Four patients were excluded as the remnant sample tested negative for LPV/r, but positive for ATV/r, indicating inaccurate clinical records in these patients. This resulted in **494** included patients.

*Table 1: Breakdown of patients in study per facility type and province*

|  | **Gauteng Province** | **Mpumalanga province** | **North-West province** | **Limpopo province** |
| --- | --- | --- | --- | --- |
| **Total patients** | ***331*** | ***79*** | ***52*** | ***32*** |
| **Primary Health Clinic** (n = 15) | ***15*** | ***7*** | ***1*** | ***2*** |
| **Community Health Centre** (n = 5) | ***11*** | ***2*** | ***-*** | ***-*** |
| **District Hospital** (n = 28) | ***57*** | ***43*** | ***5*** | ***25*** |
| **Regional Hospital** (n = 15) | ***164*** | ***13*** | ***46*** | ***-*** |
| **Provincial Tertiary Hospital** (n = 4) | ***-*** | ***14*** | ***-*** | ***5*** |
| **National Central Hospital** (n = 5) | ***84*** | ***-*** | ***-*** | ***-*** |
| **Note:** Breakdown of patients in study per facility type and province. N refers to number of facilities in dataset. One specialised tropical disease hospital was categorized as national central hospital. | | | | |

Multivariable Analysis

**Stepwise selection of variables in multivariable model:** The primary multivariable analysis presented in the manuscript included all variables that were expected to be of potential clinical relevance to the outcome: Sex, second-line NRTI backbone, log HIV-RNA load, study group (plasma versus DBS), and age. In order to test the robustness of this model, we performed a sensitivity analysis in which variables to the model were selected via stepwise backward variable selection based on Akaike information criterion (AIC). This automatic selection procedure dropped sex and second-line NRTI backbone from the model. However, the resulting adjusted odds ratios for log HIV-RNA load, study group, and age in the model did not substantially change from those presented in the main analysis.

*Table 2: Stepwise backward automatic variable selection*

| **Covariates** | **Full model** | | **Step 2** | | **Step 3** | |
| --- | --- | --- | --- | --- | --- | --- |
|  | **aOR [IQR] (p)** | **AIC*** | **aOR [IQR] (p)** | **AIC** | **aOR [IQR] (p)** | **AIC** |
| **No covariates** | **-** | **601.01** | **-** | **599.04** | **-** | **598.88** |
| **Sex**  (*female)* | *1.03 [0.68-1.56] (0.89)* | *598.96* |  |  |  |  |
| **2^nd^-line NRTI backbone** |  | *600.86* |  | *598.88* |  |  |
| ***AZT/3TC*** | *Ref* |  |  |  |  |  |
| ***TDF/3TC*** | *0.97 [0.57-1.61] (0.91)* |  | 0.97 [0.58-1.61] (0.91) |  |  |  |
| ***ABC/3TC*** | *0.65 [0.33-1.19] (0.17)* |  | 0.65 [0.34-1.19] (0.18) |  |  |  |
| ***Other*** | *0.32 [0.07-0.94] (0.07)* |  | 0.31 [0.07-0.94] (0.07) |  |  |  |
| **Log HIV-RNA at 2^nd^-line failure** *(log c/mL)* | *1.23 [0.95-1.59] (0.11)* | *601.44* | *1.21 [0.95-1.56] (0.11)* | *599.60* | *1.21 [0.95-1.56] (0.13)* | *599.20* |
| **Study group**  *(plasma)* | *2.72 [1.19-7.38] (0.03)* | *604.71* | *2.72 [1.19-7.39] (0.03)* | *602.83* | *2.65 [1.16-7.18] (0.03)* | *602.41* |
| **Age**  *(5 year increments)* | *1.21 [1.10-1.32] (<0.001)* | *615.59* | *1.20 [1.10-1.32] (<0.001)* | *614.14* | *1.21 [1.10-1.32] (<0.001)* | *614.66* |
| **Note:** 3TC = lamivudine; ABC = abacavir; AIC = Akaike information criterion; aOR = adjusted Odds Ratio; AZT = zidovudine; IQR = interquartile range; LPV/r = ritonavir-boosted lopinavir; NRTI = nucleos(t)ide reverse transcriptase inhibitor; p = p-value; TDF = tenofovir disoproxil fumarate. *AIC refers to the AIC of the model if the variable were dropped from the model. | | | | | | |

**Testing of linearity of continuous variables against the logit:** The relationship of continuous variables in the multivariable model with the predicted logit of the outcome was visualized to assess linearity. For the variable age in increments of 5 years (*Age_new*), the relationship appears linear above logit values of -2 (*figure 1A*). For the variable log HIV-RNA in copies/mL (*log_last_VL*), which is a common transformation in clinical and scientific reporting of HIV-RNA load values, the relationship is fairly linear throughout (*figure 1B*). A 10 log transformation (*Age_log*) was attempted for the age variable to evaluate if this would lead to improved linearity (*figure 1C*). The adjusted odds ratio for *Age_log* was 31.67 [IQR: 6.29-177.93]. The relationship of *Age_log* with the predicted logit was only slightly more linear. Due to this minimal improvement, and for the benefit of interpretability of the odds ratio, we opted to retain the original variable of age in increments of 5 years in the model.

*Figure 1A-C: Linearity of continuous variables against the logit*


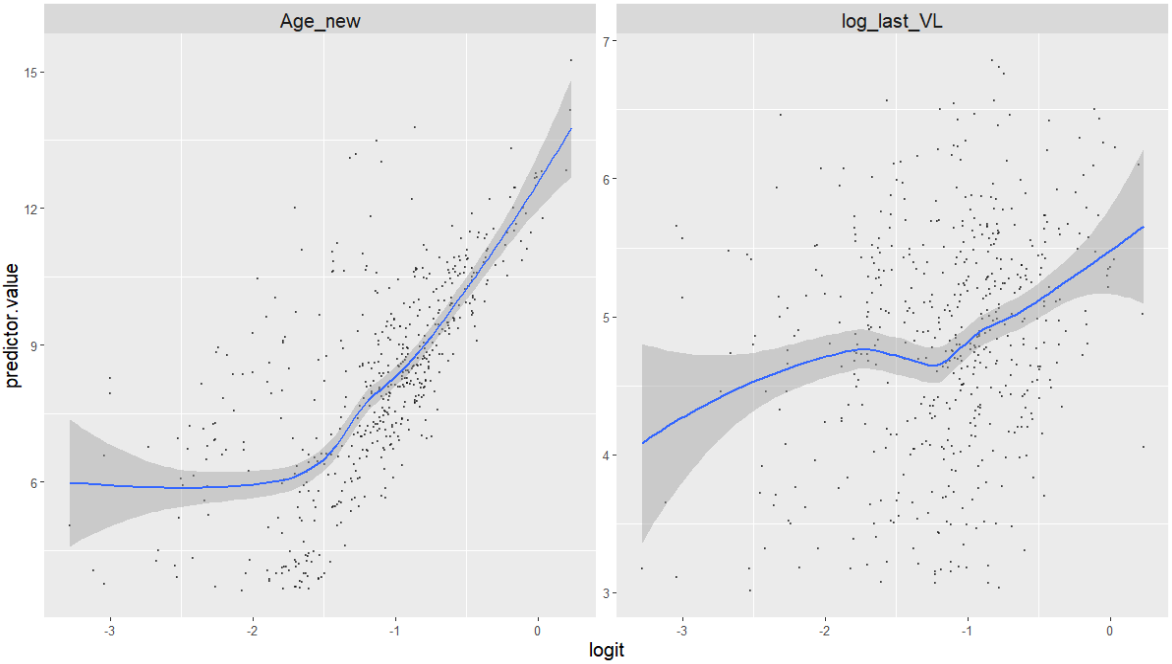


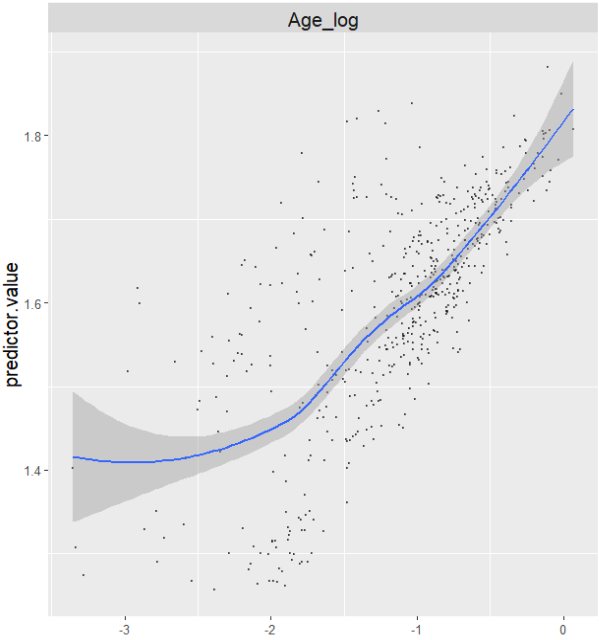


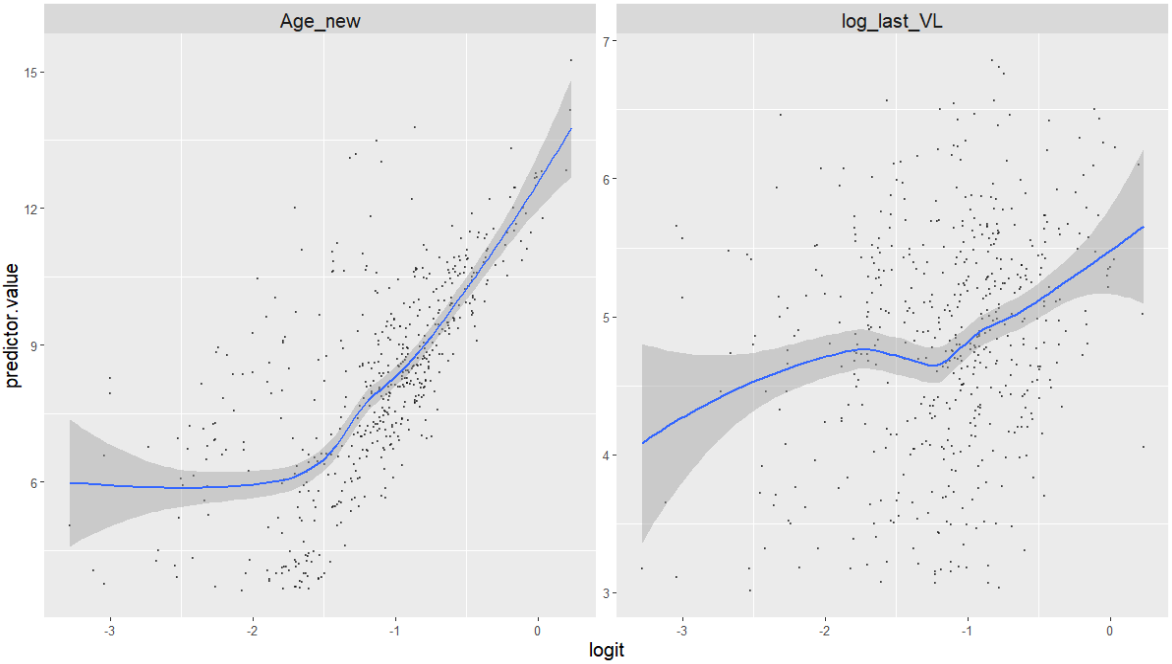

Supplement: Supplementary file 1 — Data S1. Random selection of participants in the plasma group. Table S1. Breakdown of patients in study per facility type and province. Table S2. Stepwise backward automatic variable selection. Figure S1. A‐C, Linearity of continuous variables against the logit. [file JIA2-23-e25501-s001.docx]
